# Pregnancy Outcome after Exposure to Migalastat for Fabry Disease: A Clinical Report

**DOI:** 10.1155/2019/1030259

**Published:** 2019-12-21

**Authors:** Natalja Haninger-Vacariu, Sarah El-Hadi, Udo Pauler, Marina Foretnik, Renate Kain, Zoltán Prohászka, Alice Schmidt, Nina Skuban, Jay A. Barth, Gere Sunder-Plassmann

**Affiliations:** ^1^Division of Nephrology and Dialysis, Department of Medicine III, Medical University of Vienna, Vienna, Austria; ^2^Department of Medicine I, University Hospital St. Pölten, Lower Austria, Austria; ^3^Department of Pathology, Medical University of Vienna, Vienna, Austria; ^4^Semmelweis University, Research Laboratory, Third Department of Internal Medicine, Budapest, Hungary; ^5^Amicus Therapeutics, Inc., Cranbury, NJ, USA

## Abstract

Our patient was a 37-year-old woman with Fabry disease (*GLA* p.R112H) with a medical history of recurrent headache, nausea, vomiting, vertigo, and tobacco use (20 cigarettes/day). Fabry disease was diagnosed in 2005 when she experienced proteinuria, preeclampsia, and hypertension (201/130 mm Hg) during pregnancy (delivered 50 cm, 3.4 kg healthy boy; *GLA *wild type [WT]). Enzyme replacement therapy was initiated in 2009. The patient enrolled in the phase 3 ATTRACT trial (ClinicalTrials.gov; NCT01218659) and started migalastat in May 2012 while taking hormonal contraceptives. Two years after initiating migalastat, the patient had proteinuria (2166 mg/24 h) without hypertension (131/68 mm Hg), which persisted (788 mg/24 h a month later). Kidney biopsy results were consistent with existing Fabry disease. A serum pregnancy test and ultrasound confirmed pregnancy (18 weeks' gestation). Migalastat and hormonal contraceptives were stopped; the patient continued to smoke. Fetal MRI was normal at ~29 weeks' gestation. In October 2014, at 37+ weeks' gestation, the patient delivered a 45-cm, 2.29-kg healthy girl (*GLA* WT). Excepting low birth weight, which may be related to the patient's smoking, pregnancy outcome was normal despite exposure to migalastat for 18 weeks. Migalastat therapy during pregnancy is not advised.

## 1. Introduction

Fabry disease is a rare, X-linked lysosomal storage disorder caused by deficiency of *α*-galactosidase A (*α*-Gal A), encoded by the *GLA* gene [1]. The resulting accumulation of globotriaosylceramide (GL-3) produces a wide variety of debilitating signs and symptoms, including cardiomyopathy, renal failure, cerebrovascular events, and gastrointestinal manifestations [2]. The first clinical symptoms of Fabry disease typically occur during childhood, and, if left untreated, the burden of the disease increases over time [3].

Until recently, enzyme replacement therapy (ERT), consisting of lifetime infusions of agalsidase alfa or agalsidase beta, was the only treatment approach for patients with Fabry disease [4–6]. ERT is associated with several limitations, including the burden of frequent infusions and disease progression despite adherence to therapy [7]. Migalastat is a small-molecule pharmacological chaperone designed to bind selectively and reversibly to the active sites of amenable variants of *α*-Gal A [7]. It is estimated that approximately 35%–50% of patients with Fabry disease worldwide have amenable *GLA* variants [8]. Migalastat binding stabilizes mutant forms of *α*-Gal A and facilitates trafficking to lysosomes, where dissociation of migalastat endogenous *α*-Gal A activity is restored, leading to the catabolism of GL-3 and other disease substrates [9].

In two phase 3 trials (FACETS [NCT00925301] and ATTRACT [NCT01218659]), migalastat was shown to provide clinical benefits for patients with Fabry disease and amenable *GLA* variants and was generally well tolerated [8, 10]. Migalastat is now approved for the treatment of Fabry disease in patients ≥16 years old with amenable *GLA* variants in the European Union, Switzerland, Israel, Australia, Republic of Korea, and Japan, and in adult patients in the United States and Canada [7, 9]. Because developmental toxicity was observed in rabbits who received maternally toxic doses of migalastat in preclinical studies [9] it is recommended that migalastat not be given during pregnancy. Herein, we describe the medical history and outcome of a white woman with Fabry disease who became pregnant, despite hormonal contraception, while being treated with migalastat during the phase 3 ATTRACT trial [8].

## 2. Case Presentation

### 2.1. Study Background

ATTRACT was a phase 3, randomized, open-label, active-controlled study designed to evaluate the efficacy and safety of migalastat HCl (150 mg every other day [QOD]) and ERT in patients with Fabry disease who were receiving ERT and who had an amenable *GLA* variant [8]. Full methodology is described in Hughes et al. [8]. Briefly, eligible patients were randomly assigned to either continue ERT or switch to migalastat HCl (150 mg QOD) for 18 months [8]. After completing the randomized treatment period, patients could participate in a 12-month open-label extension of migalastat HCl (150 mg QOD) [8]. Patients of reproductive potential agreed to use medically accepted methods of contraception throughout the duration of the study and for up to 30 days after the last dose of migalastat.

Written informed consent was obtained from all study participants before initiating any study-related procedures. The appropriate ethics committee approved the clinical study protocol at each site and the trial was conducted in accordance with the Declaration of Helsinski.

All clinical chemistry analyses were performed in an International Standardization Organization (ISO) 15189 accredited clinical laboratory at the Department of Laboratory Medicine, Medical University of Vienna. Glomerular filtration rate was estimated (eGFR) using the Chronic Kidney Disease Epidemiology Collaboration (CKD-EPI) equation. Analyses of complement-related proteins was performed at the third Department of Internal Medicine, Semmelweis University, Budapest, Hungary. Genetic testing for rare variants in *GLA* and complement-related genes was performed as previously described [11].

### 2.2. Patient History

**Figure 1:**
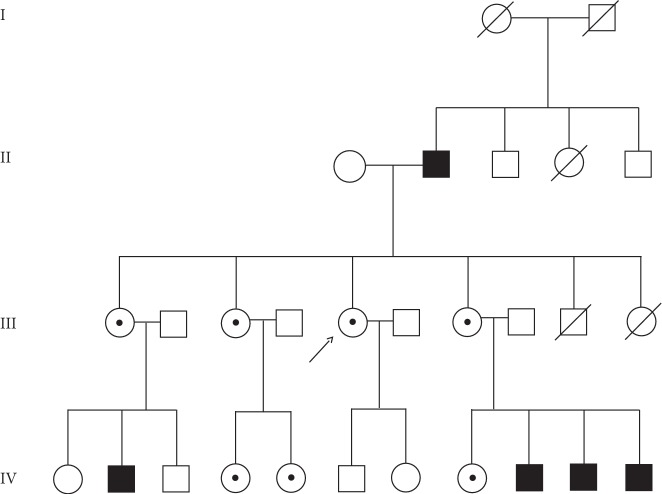
Pedigree analysis. The patient is indicated by the arrow. Black boxes represent males with Fabry disease; circles with black dots represent females with Fabry disease. Slash indicates deceased.

**Figure 2:**
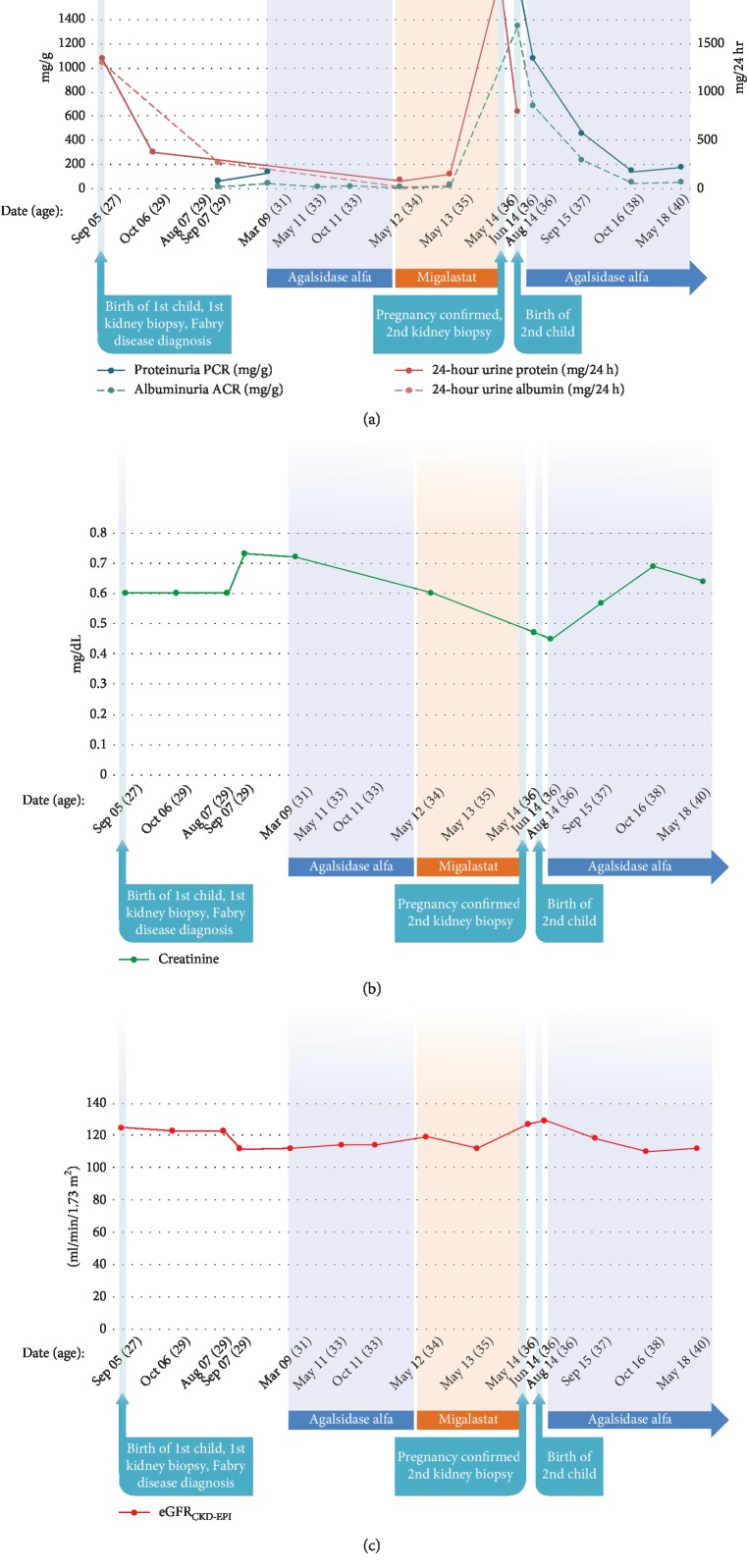
Time course of (a) proteinuria and albuminuria, (b) creatinine, and (c) eGFR_CKD-EPI_. ACR, albumin-to-creatinine ratio; eGFR_CKD-EPI_, estimated glomerular filtration rate chronic kidney disease epidemiology collaboration; PCR, protein-to-creatinine ratio.

**Figure 3:**
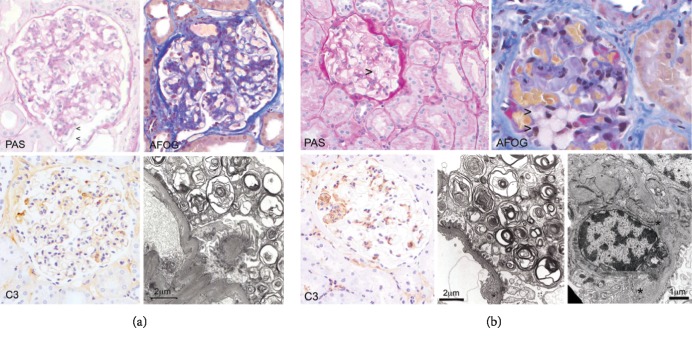
Histology and transmission electron microscopy of kidney biopsy specimens obtained (a) in 2005, one month after birth of first child (second pregnancy) and (b) in 2014, during pregnancy with second child (third pregnancy). AFOG, acid fuchsin orange G; IgA, immunoglobulin A; IgG, immunoglobulin G; PAS, periodic acid-Schiff; TEM, transmission electron microscopy. (a) The first renal biopsy (2005) shows a diffuse, segmentally accentuated mesangial matrix and mesangial cell proliferation (PAS and AFOG) in >50% of the glomeruli, and segmentally obliterated capillary loops adherent to the Bowman's capsule (AFOG). There is dominant segmental C3 deposition (C3) in the absence of IgG and IgA. Podocytes show characteristic lamellar and zebroid bodies by TEM; however, the classical appearance of “foamy” podocytes was less dominant by light microscopy (PAS) due to the segmental nature of the pathological changes. (b) The second renal biopsy (2014) shows characteristic foamy macrophages (PAS and AFOG) as well as segmental mesangial matrix and mesangial cell proliferation. Dominant C3 deposits (C3) correspond to mesangial electron dense deposits (∗) by TEM (right) while almost all podocytes contain lamellar and zebroid inclusion bodies (a).

The patient is a white woman who was diagnosed with Fabry disease in 2005 at 28 years of age.****Her father had Fabry disease (generation II), and all 3 surviving siblings (all females) also had Fabry disease (generation III) (Figure 1). The patient's medical history prior to migalastat treatment was significant for headache (beginning at 12 years of age ), psoriasis (beginning at 17 years of age), and nausea, vomiting, and vertigo (beginning at 18 years of age). The patient had a history of smoking (since the age of 14) and smoked 20 cigarettes per day. Her first pregnancy, which occurred in 2002 at the age of 25, resulted in a miscarriage during the fifth month (18 weeks' gestation) after having a traffic accident (Table 1). At 26 years of age, the patient was hospitalized for strong headache and neck pain. At 27, the patient had a second pregnancy. An emergency cesarean delivery was performed at 38+ weeks of gestation due to preeclampsia (blood pressure ≥140/90 mm Hg), proteinuria (urine test for proteinuria was positive), and pelvic abnormalities, which resulted in delivery of a healthy male infant (50 cm, 3.4 kg, *GLA* WT) (Table 1). Twenty days postpartum, an increase in 24-hour urine protein (935.9 mg/24 h) was recorded. Then, 1-month postpartum, an increase in 24-hour urine protein (1340 mg/24 h, Figure 2), hypertension (210/130 mm Hg), severe neck pain, headache, and edema resulted in a 7-day hospital stay and consultation with a nephrologist. During this time, Fabry disease was diagnosed based on kidney biopsy results (Figure 3(a)) and mutational analysis (*GLA* p.R1112H). For the next 4 years, the patient received no specific Fabry disease treatment, and experienced the occasional occurrence of leg edema and moderate swelling. In March 2009, at the age of 31, the patient experienced a mild increase of proteinuria (from 59 mg/g in September 2007 to 132 mg/g in March 2009) and headaches and was diagnosed with depression. The patient began treatment with agalsidase alfa via infusion; during therapy, there was no progression of renal, cardiac, or neurological symptoms. Two years later, in 2011, the patient experienced new symptoms of chronic pharyngitis (secondary to tobacco use) and euthyroid goiter (thyroid volume, 31.8 mL). Ongoing psoriasis was treated with psoralen and long-wave ultraviolet radiation (PUVA) phototherapy.

### 2.3. Migalastat Treatment and Pregnancy

In May 2012, the patient enrolled in the ATTRACT trial and was randomly assigned to migalastat HCl (150 mg QOD) [8]. At enrollment, 24-h urine protein was 83 mg/24 h. In accordance with the study protocol, the patient agreed to use hormonal contraceptives (Diane mite; ovulation inhibitor) for the duration of the study. While enrolled in the study, renal, cardiac, and neurologic assessments conducted through February 2014 showed no evidence of disease progression. In May 2014, after 2 years of migalastat treatment, proteinuria increased sharply from 78 mg/24 h (in February 2014) to 2166 mg/24 h, without hypertension (131/68 mm Hg). A urine pregnancy test was negative. The following month, ongoing proteinuria (788 mg/24 h, Figure 2) prompted kidney biopsy, which showed cellular characteristics consistent with existing Fabry disease (Figure 3(b)). A serum pregnancy test result was positive, and pregnancy was confirmed via ultrasound examination (18 + 0 weeks gestation). Migalastat therapy and hormonal contraceptives were stopped; the patient continued to smoke during her pregnancy (20 cigarettes/day).

**Figure 4:**
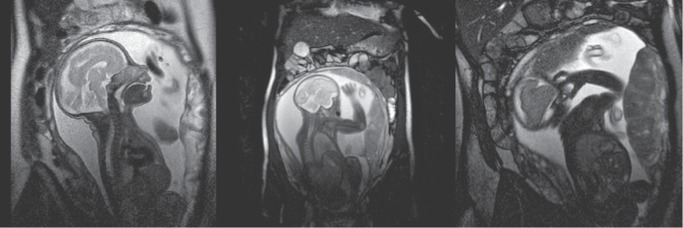
Fetal MRI (coronal plane) during pregnancy at 29 weeks' gestation. MRI, magnetic resonance imaging. Images presented with patient permission.

At 29 weeks' gestation, magnetic resonance imaging indicated normal fetal development (Figure 4). The pregnancy was uneventful, and a healthy female infant (45 cm, 2.29 kg, *GLA* WT, Table 1) was delivered via cesarean section at 37+ weeks of gestation. After delivery, ERT therapy (agalsidase alfa, home infusion) was restarted and is currently ongoing.

Because the patient had complement C3 staining in her kidney biopsy specimens (Figure 3), serologic and genetic complement analyses were performed. The complement system is an important mediator of kidney injury, and complement activation is associated with a wide variety of kidney diseases [12]. Results showed that the patient had a normal complement profile, was anti-C1q, anti-FH, and C3Nef negative, and had no signs of complement activation or consumption. DNA sequencing revealed no mutations in *CFH*, *CFI*, *CD62*, *C3*, *CFB*, *THBD*, *CFHR5*, and *ADAMTS13*; MLPA results showed no disease-causing deletions or duplications of *CFH*, *CFHR1*, *CFHR2*, *CFHR3*, and *CFHR5*.

## 3. Discussion

We report on the case of a woman with Fabry disease who had a successful pregnancy outcome after receiving migalastat for 18 weeks, with no significant negative effect on her child. Except for a birth weight (2.29 kg) that met criteria for being low for gestational age [13, 14], the pregnancy outcome in this case was normal. The specific reason for the low birth weight is unknown; however, the patient smoked during her pregnancy, which is known to be associated with low birth weight [15, 16]. To our knowledge, there is no report of an association between low birth weight and Fabry disease and normal birth weight has been reported for Fabry-complicated pregnancies [17].

Interestingly, both kidney biopsies (one obtained shortly after her second pregnancy, and one obtained during the third pregnancy) showed evidence of Fabry disease and segmental C3 staining in the glomeruli. In-depth serological and genetic analyses did not confirm a specific cause for C3 glomerulopathy. Thus, our findings may point to an inflammatory response in the kidney triggered by pregnancy and Fabry disease, resulting in heavy proteinuria during pregnancy that resolved after childbirth in both instances.

Successful pregnancy outcomes have been reported in women with Fabry disease receiving ERT with agalsidase alfa [18–20] or agalsidase beta [20–25] infusions. This is the first report of a successful pregnancy outcome with migalastat. In a separate phase 3 trial of migalastat in patients with Fabry disease [10] one woman discontinued from the study because of pregnancy after being exposed to migalastat for 34 days. The pregnancy was uneventful, and she delivered a healthy baby girl. In a rabbit embryo-fetal toxicity study, migalastat doses associated with maternal toxicity were associated with embryo-fetal death, a reduction in mean fetal weight, retarded ossification, and slightly increased incidence of minor skeletal abnormalities [9]. Therefore, migalastat therapy is not advised during pregnancy. Despite the successful pregnancy reported here, the safety of migalastat during pregnancy is still uncertain.

## Figures and Tables

**Table 1:** Overview of pregnancies.

Pregnancy	Year of delivery	Age of mother at delivery (years)	Outcome	Sex of child	Gestational age at birth (weeks)	Mode of delivery	Birth weight (kg)	Birth height (cm)	Head circumference at birth (cm)	Assessment^a^	*GLA*
1	2002	25	Miscarriage	N/A	±18	Vaginal	N/A	N/A	N/A	N/A	N/A
2	2005	27	Live birth	Male	38 + 6	Emergency C-section	3.40	50	33.3	AGA	WT
3	2014	37	Live birth	Female	37 + 1	C-section	2.29	45	32	SGA	WT

AGA, appropriate gestational age; N/A, not available; SGA, small for gestational age; WHO, World Health Organization; WT, wild type.

^a^According to WHO child growth standards. SGA is defined as infants with a birth weight below the 10th percentile for gestational age [26].
